# *Shewanella putrefaciens*: a rare cause of purulent otorrhoea

**DOI:** 10.1099/acmi.0.000469.v3

**Published:** 2022-12-07

**Authors:** Elmostafa Benaissa, Adil Maleb, Mostafa Elouennass

**Affiliations:** ^1^​ Epidemiology and Bacterial Resistance Research Team/BIO-INOVA Centre, Faculty of Medicine and Pharmacy, University Mohammed V, Rabat, Morocco; ^2^​ Department of Bacteriology, Mohammed V Military Teaching Hospital, Faculty of Medicine and Pharmacy, University Mohammed V, Rabat, Morocco; ^3^​ Laboratory of Microbiology, Mohammed VI University Hospital, Faculty of Medicine and Pharmacy, University Mohammed I, Oujda, Morocco

**Keywords:** *Shewanella putrefaciens*, otorrhoea, purulent

## Abstract

*
Shewanella putrefaciens* is a Gram-negative, non-fermenting, motile and oxidase-positive bacillus. Its incrimination in human pathology is very rare, although there has been a resurgence in *Shewanella* infections in recent years. We report the first case in Morocco of a purulent otorrhoea caused by *S. putrefaciens *, resistant to conventional treatment, occurring in a 25-year-old female, afebrile, without deterioration of the general state and possibly acquired during sea bathing. We also describe the bacteriological characteristics of and antibiotic susceptibility results for the isolate.

## Data Summary

No data was reused or generated.

## Introduction

The genus *Shewanella* is in the family *Shewanellaceae *, which includes Gram-negative, non-fermentative, motile and oxidase-positive bacilli. *Shewanella putrefaciens* was formerly known as *Pseudomonas putrefaciens*; it was classified in the family *Vibrionacae* until the 1990s, when it was reclassified into the genus *Shewanella* [1]. The majority of these bacteria were originally found in aquatic environments [2].

Human infections are rare, but have been increasingly reported in recent years, including bacteraemia and skin and soft tissue infections [3]. However, purulent otorrhoea due to *S. putrefaciens* is very rare. We report the first case in Morocco of a purulent otorrhoea caused by *S. putrefaciens *, resistant to conventional treatment, occurring in a 25-year-old female, afebrile, without deterioration of the general state and possibly acquired during sea bathing. We also describe the bacteriological characteristics of and antibiotic susceptibility results for the isolate.

## Case report

The patient is a 25-year-old woman, without any medical history, treated in our ENT department for chronic otitis media with open eardrum. Her ear had been dry for more than 6 months after treatment including amoxicillin–clavulanic acid 3 g/day associated with ofloxacin ear drops. Currently, she has been admitted for purulent foetid otorrhoea following a swim at the beach. The illness is 1 month old. The symptomatology started 4 days after her swim, motivating her consultation with a general physician who prescribed the same probabilistic treatment as before, based on amoxicillin–clavulanic acid 3 g/day associated with ofloxacin ear drops for 15 days without improvement. Clinical examination on admission was normal. Blood tests showed a CRP of 150 mg l^−1^ and hyperleukocytosis of 12 000 GB ml^−1^.

As part of the aetiological exploration, an auricular pus sample was taken in order to identify the germ responsible for the infection and adapt the treatment. Direct microscopic examination of the pus showed numerous neutrophils, a few epithelial cells and numerous Gram-negative bacilli. Pus was cultured in Columbia agar, polyvitex chocolate agar, Columbia agar with nalidixic acid, nystatin and colistin, and Sabouraud agar with chloramphenicol. Cultures were placed in an incubator supplemented with 5–10 % CO_2_ for 24 h at 37 °C. All cultures were pure with pigmented colonies, showing no haemolysis on Columbia agar after 2 days of incubation. Colonies were oxidase-positive. Identification was performed using an API 20NE gallery (bioMérieux, Marcy l'étoile, France), resulting in an excellent identification (code 3051354) of the *S *. *putrefaciens* group. However, the API 20NE gallery cannot distinguish between *Shewanella algae* and *S. putrefaciens *. Therefore, we required additional tests based on the criteria of Nozue *et al*. [4] for better species identification (see Table 1).

**Table 1.** The criteria of Nozue *et al*. [4] for the identification of *S. putrefaciens
*

Bacteriological and biochemical properties	Results
Gram staining	Gram-negative bacilli
Oxidase test	+
Colonies	Pigmented
Haemolysis on Columbia agar	–
Growth at or on:	
4 °C	+
Environmental temperature:	+
42 °C	–
*Salmonella–Shigella* agar	–
Acid production from:	
Maltose	+
Glucose	+
Saccharose	+
Arabinose	+

Antibiotic susceptibility testing was performed using the microdilution method with Sensititre Gram-negative MIC plates (Thermo Scientific, France) from a young culture. Results were interpreted in accordance with the Comité De l'Antibiogramme de la Société Française de MicrobiologieCASFM (CASFM)/European Committee on Antimicrobial Susceptibility Testing (EUCAST) 2021 recommendations.

Our isolate was sensitive to all antibiotics tested except amoxicillin and amoxicillin–clavulanic acid (Table 2).

**Table 2.** Antibiotic sensitivity of the *S. putrefaciens* isolate

Antibiotics	* Shewanella putrefaciens *	
	MIC (µg ml^−1^)	Clinical categorization
Amikacine	<4	Sensitive
Aztreonam	2	Sensitive
Cefepime	<2	Sensitive
Cefotaxim	<1	Sensitive
Ceftazidim	<1	Sensitive
Ciprofloxacin	<0.25	Sensitive
Colistin	<0.25	Sensitive
Doripenem	<0.12	Sensitive
Doxycyclin	<2	Sensitive
Ertapenem	<0.25	Sensitive
Gentamicin	<1	Sensitive
Imipenem	<1	Sensitive
Levofloxacin	<1	Sensitive
Meropenem	<1	Sensitive
Minocyclin	<2	Sensitive
Piperacillin–tazobactam 4	<8/4	Sensitive
Polymixin B	<0.25	Sensitive
Ticarcillin–clavulanic acid 2	<16/2	Sensitive
Tigecycline	0.25	Sensitive
Tobramycin	<1	Sensitive
Trimethoprim–sulfamethoxazole	<0.5/9.5	Sensitive

The patient was placed on levofloxacin 500 mg twice a day for 15 days with a good clinical evolution marked by the interruption of purulent discharge after 10 days of treatment.

## Discussion

*Shewanella* spp. are Gram-negative bacilli found in seawater [5]. They allow a renewal of organic matter and also a reduction of various metals and substances [1]. The *Shewanella* spp existing in clinical samples are *S. putrefaciens* and *S. algae *, and currently more than 80 % of human isolates are *S. algae* [4, 6–8]. At present, there are molecular methods based on 16S rRNA and *gyrB* to differentiate *S. algae* from *S. putrefaciens *, but these tests are used more in the research laboratory than in the routine laboratory. However, these two species can easily be distinguished by phenotypic properties such as the capacity of *S. algae* to develop at 42 °C and a high concentration of NaCl (e.g. 6 %), unlike *S. putrefaciens *. In a study reported by Holt *et al*., of 164 clinical isolates from ear samples, 5 isolates were identified as *S. putrefaciens* [9]. Another case of S. *putrefaciens* isolated from an ear swab was reported by Martín-Rodríguez *et al*. [10]. Our case represented the seventh case of *S. putrefaciens* otitis in the literature and the second in our Mediterranean region. Using molecular methods, other species have been reported in the literature, such as *Shewanella chilikensis *, *Shewanella carasii* and *Shewanella xiamenensis* [11–13] .

These *Shewanella* infections have been reported in coastal areas and hot climates. In recent years, cases have begun to occur in temperate regions [6, 14, 15]. These infections are frequent in July, August and September, with a few cases in October [1]. Our case occurred inSeptember, which is in accordance with the literature.

Contact with seawater remains the most common source of human infection. Cases with contact with seawater have been reported in numerous studies [9, 14, 16–20] and in another Danish study on ear infections, where more than 80 % of the patients had some exposure [6]. Our case supports the data in the literature. *Shewanella* infections predominantly occur among males, although this predominance may be due to genetic or sociocultural factors [10]. Skin and tissue infections following skin tears or trauma are the clinical syndrome most described in the literature [14, 18–25]. According to a Danish study, most patients present with symptoms of acute or chronic otitis or non-specific ear discharge and are between 3 and 15 years of age [6] . On the other hand, our patient was 25 years old and had a chronic otitis with purulent discharge.

Holt *et al*. reported that the time to onset of symptoms varies between 1 to 5 days after exposure to seawater [6]. Our patient reported that she had a discharge 4 days after swimming in the beach. According to the same Danish study cited above, 49 % of *S. algae* isolates were isolated in pure cultures, documenting the pathogenic potential of this emerging germ [6]. Our strain has been identified in pure culture.

*S. algae* and *S. putrefaciens* retain good sensitivity to aminoglycosides, carbapenems, erythromycin and quinolones except penicillin [6, 8, 9, 20, 26, 27]. These isolates have variable susceptibility to amoxicillin and cephalosporins, with more isolates susceptible to third- and fourth-generation cephalosporins than to first- and second-generation cephalosporins [6, 9, 26, 27]. In a Danish study, all *S. algae* isolates were susceptible to piperacillin, aminoglycosides, ciprofloxacin, erythromycin and tetracycline, with variable susceptibility to ampicillin and cephalosporins [9]. Holt *et al*. reported that all isolated *S. algae* showed resistance to colistin, and six *S. putrefaciens* isolates were susceptible [6]. Therefore, polymyxin sensitivity can be used to differentiate between the two species. Our *S. putrefaciens* isolate was susceptible to all antibiotics tested except amoxicillin and amoxcillin–clavulanic acid. According to the literature, the treatment of *Shewanella* infections is easy and includes surgical and medical treatment [6, 9, 14, 19, 25, 28, 29]. Medical treatment is based on β-lactams, aminoglycosides and quinolones, provided that the strain is sensitive to these molecules [1].

## Conclusion

The emergence of *Shewanella* infections in our region requires us to consider them in patients with a history of ear disease and recent contact with seawater.
